# Dichotic Perception of Lexical Tones in Cantonese-Speaking Congenital Amusics

**DOI:** 10.3389/fpsyg.2020.01411

**Published:** 2020-07-07

**Authors:** Jing Shao, Caicai Zhang

**Affiliations:** ^1^School of Humanities, Shanghai Jiao Tong University, Shanghai, China; ^2^Shenzhen Institutes of Advanced Technology, Chinese Academy of Sciences, Shenzhen, China; ^3^Department of Chinese and Bilingual Studies, The Hong Kong Polytechnic University, Hong Kong, China; ^4^Research Centre for Language, Cognition, and Neuroscience, The Hong Kong Polytechnic University, Hong Kong, China

**Keywords:** congenital amusia, dichotic listening, ear preference, lexical tone perception, Cantonese

## Abstract

Congenital amusia is an inborn neurogenetic disorder of musical pitch processing, which also induces impairment in lexical tone perception. However, it has not been examined before how the brain specialization of lexical tone perception is affected in amusics. The current study adopted the dichotic listening paradigm to examine this issue, testing 18 Cantonese-speaking amusics and 18 matched controls on pitch/lexical tone identification and discrimination in three conditions: non-speech tone, low syllable variation, and high syllable variation. For typical listeners, the discrimination accuracy was higher with shorter RT in the left ear regardless of the stimulus types, suggesting a left-ear advantage in discrimination. When the demand of phonological processing increased, as in the identification task, shorter RT was still obtained in the left ear, however, the identification accuracy revealed a bilateral pattern. Taken together, the results of the identification task revealed a reduced LEA or a shift from the right hemisphere to bilateral processing in identification. Amusics exhibited overall poorer performance in both identification and discrimination tasks, indicating that pitch/lexical tone processing in dichotic listening settings was impaired, but there was no evidence that amusics showed different ear preference from controls. These findings provided temporary evidence that although amusics demonstrate deficient neural mechanisms of pitch/lexical tone processing, their ear preference patterns might not be affected. These results broadened the understanding of the nature of pitch and lexical tone processing deficiencies in amusia.

## Introduction

Like impairment in language is known as “aphasia,” impairment in music perception and production is known as “amusia.” Congenital amusia (amusia hereafter) is a deficit of fine-grained pitch processing, which has a negative influence on mistuned tone detection and out-of-key tone detection (Ayotte et al., 2002). Amusia occurs in about 1.5–4% of the population (Peretz and Hyde, 2003; Peretz and Vuvan, 2017).

Earlier research showed that amusia is primarily a pitch deficit (Peretz et al., 2002). This is because despite suffering from severe musical impairment in daily life, individuals with amusia were rarely reported to have severe deficits in everyday communication. For instance, amusics have little difficulty in the recognition and perception of intonation which involves large pitch differences and with aided linguistic cues (Ayotte et al., 2002). However, when the pitch difference was tuned to be small, a series of studies have shown that amusics performed worse than musically intact controls in processing speech intonation and emotion prosody (Jiang et al., 2010, 2012a; Liu et al., 2010; Thompson et al., 2012; Lu et al., 2015), suggesting that amusia influences speech processing negatively.

In addition to intonation and emotion prosody processing, a wide range of studies have reported inferior performance of amusics in lexical tone perception (Nan et al., 2010; Tillmann et al., 2011b; Jiang et al., 2012b; Liu et al., 2012; Wang and Peng, 2014; Shao et al., 2016, 2019; Zhang et al., 2017b, 2018). There is evidence that both low-level phonetic processing and high-level phonological processing of lexical tones were impaired. Firstly, speakers with amusia were less accurate at the discrimination of lexical tones, which is often thought to reflect a relatively low level of phonetic processing. For example, French speakers with amusia (Nguyen et al., 2009; Tillmann et al., 2011a) showed inferior performance in the discrimination of non-native Mandarin and Thai tones. Since these French participants were naïve to Mandarin and Thai tones, the perception of tones was deemed to mainly reflect the phonetic processing abilities. For tonal language speakers with amusia, under the conditions where perception primarily relied on the acoustic comparison of two tones, i.e., discrimination of lexical tones that were carried by the same syllables, a similar finding was reported. Amusics who are native tonal-language speakers often showed degraded performance, suggesting that their phonetic processing of lexical tones was impaired (Liu et al., 2012; Shao et al., 2016, 2019).

On the other hand, using designs that measure lexical tone processing at a more abstract level, another line of research showed that native tonal-language speakers with amusia were also impaired in the phonological processing of lexical tones. Part of the evidence for this claim rises from the impaired categorical perception of native tones (Jiang et al., 2012b; Huang et al., 2015; Zhang et al., 2017b). For example, Mandarin-speaking amusics failed to obtain higher discrimination accuracy on the between-category stimuli than the within-category stimuli, suggesting a lack of categorical perception of lexical tones (Jiang et al., 2012b). In addition, studies which manipulated the degree of acoustic variation in lexical tone perception also demonstrated that amusics have difficulty in abstracting tone categories in the acoustically more variable context (Nan et al., 2010; Shao et al., 2019). For instance, discriminating a pair of tones that were carried by different base syllables involves breaking down the syllable and extracting tone categories, which is thought to measure phonological processing of lexical tones (Shao et al., 2019). Amusics demonstrated degraded performance in such tasks, suggesting that high-level phonological processing of lexical tones was also impaired in amusics.

Taken together, impairments related to lexical tone perception in amusia have been found in both phonetic and phonological processing levels. However, an important yet under-studied question is the ear advantage/hemispheric specialization of pitch and lexical tone perception. Plenty of studies have shown that the left hemisphere (LH) is more specialized at linguistic/verbal processing, such as the processing of spoken words, syllables, and digits (Kimura, 1967; Studdert-Kennedy and Shankweiler, 1970; Hugdahl et al., 1999), as revealed by a right ear advantage (REA) in the dichotic listening task; whereas the right hemisphere (RH) is more dominant in musical/non-verbal processing, such as intensity judgments (Brancucci et al., 2008), timbre detection (Hugdahl et al., 1999), pitch discrimination (Wioland et al., 1999), and musical structure processing (Hoch and Tillmann, 2010), as revealed by a left ear advantage (LEA). To the best of our knowledge, the ear preference in lexical tone perception in amusia has never been examined before. Thus, whether and how the deficits in phonetic and phonological processing levels would influence the ear advantage of lexical tone perception in amusia remains an open question.

Previous dichotic listening studies have generally observed an REA or bilateral pattern in processing lexical tones for native tonal language speakers (Van Lancker and Fromkin, 1973, 1978; Baudoin-Chial, 1986; Moen, 1993; Wang et al., 2001). For example, Van Lancker and Fromkin (1973) found that native speakers of Thai showed a significant REA for both tones and consonants, but no ear preference for non-speech hums; in contrast, English speakers only showed an REA for the consonants, but no ear preference for either Thai tones or hums. These findings were confirmed by a follow-up study (Van Lancker and Fromkin, 1978), which explored whether the REA found in the Thai speakers was caused by their native language knowledge or general familiarity with pitch differences. The results showed that native speakers of Thai, English-speaking musicians, and English-speaking non-musicians all exhibited the REA for consonants, but only Thai speakers had the REA for lexical tones. Similar findings were reported with native Mandarin Chinese (Wang et al., 2001) and Norwegian speakers (Moen, 1993). Note that one study actually reported bilateral processing of lexical tones in native Mandarin Chinese listeners, who showed no ear preference in dichotic perception, similar to the pattern of consonants and hums (Baudoin-Chial, 1986).

However, a recent study on Cantonese revealed a consistent LEA/RH specialization in the perception of lexical tones in native Hong Kong Cantonese speakers (Jia et al., 2013). The authors presented Cantonese level and contour tones in separate blocks in an identification task as well as a discrimination task. Three types of stimuli were designed, including hums, pseudo-syllables, and real syllables. The results showed a consistent LEA of lexical tone processing in Cantonese speakers, regardless of the tone type, stimulus type, and task. The authors attributed the discrepancy in ear preference patterns between Cantonese and other tonal languages to the specificity of the Cantonese tonal system. As the Cantonese tonal space is much denser, with a total of nine tones in contrast to four tones in Mandarin, the perception of Cantonese tones might rely more on refined acoustic differences, resulting in a greater LEA.

It is worth noting that although common findings from dichotic listening studies suggested an REA or bilateral pattern for lexical tone processing in tonal language speakers (with the exception of Hong Kong Cantonese, which showed an LEA) (Van Lancker and Fromkin, 1973, 1978; Moen, 1993; Wang et al., 2001; Jia et al., 2013), a more recent study revealed that the ear preference of lexical tone perception in dichotic listening may not only be modulated by native language experience, but may also be influenced by speech content (Mei et al., 2020). The authors found that when hummed tones that only included slow frequency modulations of tones were used as stimuli, an LEA was found. However, when different levels of phonological and linguistic content were incrementally added in the stimuli, the LEA became unstable and more LH participated, resulting in more bilateral processing of lexical tones. Incorporating the findings from Wang et al. (2001) and Mei et al. (2020) concluded that the ear preference shifted from LEA to bilateral processing and finally REA with the phonological and lexical-semantic attributes gradually added.

As mentioned above, there remains an important gap in research on amusia about how the deficits of amusics in phonetic and phonological processing interfere with the brain lateralization of lexical tone perception. Based on the previous findings (Van Lancker and Fromkin, 1973, 1978; Baudoin-Chial, 1986; Moen, 1993; Wang et al., 2001; Jia et al., 2013; Mei et al., 2020), we speculated that the impairment in low-level phonetic/acoustic and high-level phonological processing of lexical tones observed in amusics may interfere with the ear preference patterns in different conditions that require different levels of speech processing.

To this end, we examined how deficits in phonetic and phonological processing in amusia affect the brain specialization of lexical tone perception in dichotic listening settings under three stimulus conditions: non-speech tone, low syllable variation, and high syllable variation. The three conditions were designed to measure low to high levels of processing (auditory, phonetic, and phonological processing) in the dichotic setting. In the non-speech tone condition, where the stimuli were pure tone sounds, perception in this condition mainly reflects the auditory/acoustic processing of the pitch information. In the low and high variation conditions, the stimuli were meaningful Cantonese words, where the critical difference was that the carrying syllables were always the same in a dichotic pair in the low variation condition, but always different in the high variation condition. Perception in the low variation condition is thought to reveal more phonetic processing of lexical tones, but in the high variation condition, abstracting tone categories from the spoken words is thought to measure the relatively higher phonological processing of lexical tones in dichotic listening settings.

For typical listeners, according to previous findings on tonal language speakers (Luo et al., 2006; Mei et al., 2020), it is hypothesized that in the non-speech tone condition where acoustic details may be the primary cue, an LEA would be observed. In the low variation condition, real syllables were used but with no syllable variation within each trial; according to Mei et al. (2020), which found that the LEA started to become unstable with increased speech content, it is predicted that the LEA would become less prominent compared to the non-speech tone condition. Finally, in the high variation condition, where more phonological/linguistic computing is demanded, a further reduced LEA or bilateral processing/REA would be expected.

For amusics, since they are reported to be impaired in auditory processing of non-speech pitch and phonetic and phonological processing of lexical tones (Zhang et al., 2017b; Shao et al., 2019), we anticipate lower accuracy in amusics in pitch and lexical tone perception in all conditions. As for the ear preference and how it will be affected in amusia, this remains an open question, although some speculations could be made based on the previously reported neural deficits in amusia. It has been found that neural impairments in amusia implicate a RH frontotemporal network including the inferior frontal gyrus (IFG) and superior temporal gyrus (STG) (Hyde et al., 2011; Albouy et al., 2013; Chen and Yuan, 2016; Wang et al., 2017; Zhang et al., 2017a). In addition, abnormal lack of activation in the right STG during lexical tone perception has been found in Cantonese speakers with amusia (Zhang et al., 2017a). These impairments might hinder the RH involvement in the pure tone and low variation condition, leading to reduced LEA or less stable LEA in these conditions. In the high variation condition, as amusics have been found to be impaired in accessing phonological representations compared to typical controls (Jiang et al., 2012b; Zhang et al., 2017b), they may be less able to recruit the LH, rendering the bilateral processing/REA in this condition less clear. However, an alternative possibility is that as *native* speakers of Cantonese, amusics might show more or less comparable ear preference patterns to typical controls (in contrast to *non-native* listeners who typically exhibited the LEA in lexical tone perception; Van Lancker and Fromkin, 1973, 1978; Moen, 1993; Wang et al., 2001; Mei et al., 2020), albeit exhibiting overall lower accuracy than controls. This possibility is supported by findings that amusics demonstrate more or less normal pitch and lexical tone perception in certain listening conditions such as when the pitch differences are large or when no focal attention to the stimuli is required (Peretz et al., 2005; Moreau et al., 2013; Zendel et al., 2015; Zhang and Shao, 2018); additionally, amusics retained some abilities of phonological processing, despite having worse performance than controls (Zhang et al., 2018; Shao et al., 2019). If so, we predicted that Cantonese-speaking amusics would exhibit an overall degraded performance in dichotic perception of lexical tones, but showing similar ear preference patterns as controls.

## Materials and Methods

### Participants

Eighteen amusics and 18 musically intact controls participated in this experiment. They were all native speakers of Hong Kong Cantonese. The participants were pre-screened based on the criteria of being right-handed, with no hearing impairment, and with no history of formal musical training. Amusics and controls were identified using the Montreal Battery of Evaluation of Amusia (MBEA) (Peretz et al., 2003). The MBEA consists of six subtests: *scale*, *contour* and *interval* are pitch-based tests, *rhythm* and *meter* are duration-based tests, and the last one is a *memory* test. All amusic participants scored below 71% (Nan et al., 2010) in the global score, which is the mean of all six subtests, whereas all control participants scored higher than 80%. Control participants were matched with amusic participants one by one in age, gender, and years of education. Demographic characteristics of the participants are summarized in Table 1. The experimental procedures were approved by the Human Subjects Ethics Sub-committee of The Hong Kong Polytechnic University. Informed written consent was obtained from participants in compliance with the experiment protocols.

**TABLE 1:** Demographic characteristics of the amusic and control participants.

Subject information	Amusics	Controls
No. of participants	18 (8 M, 10 F)	18 (8 M, 10 F)
Age (range)	22.35 ± 2.8 years	22.5 ± 3.1 years
	(19.1–27.5 years)	(18.7–28.5 years)
*MBEA (SD)*		
Scale	50.4 (16.1)	89.9 (5.9)
Contour	54.6 (16.9)	90.2 (5.5)
Interval	50.4 (17.2)	91.7 (4.6)
Rhythm	53.9 (16.9)	93.1 (4.7)
Meter	47.2 (12.4)	75.7 (16.1)
Memory	62.4 (24.2)	97.9 (2.3)
Global	53.1 (14.5)	89.8 (3.4)

*Amusics and controls were identified using the Montreal Battery of Evaluation of Amusia (MBEA) (Peretz et al., 2003). Amusics scored lower than 71% in the global score, which is the mean of all six subtests, whereas controls scored higher than 80%. M = male; F = female.*

### Stimuli

There were three types of stimuli in the current study: non-speech tone, low variation, and high variation conditions. The target stimuli used in the low variation condition were six words contrasting six Cantonese tones on the syllable /ji/: high level tone (T1), high rising tone (T2), mid level tone (T3), extra low level/low falling tone (T4), low rising tone (T5), and low level tone (T6) (see Table 2; Bauer and Benedict, 2011; Matthews and Yip, 2013). The target stimuli used in the high variation condition were 18 meaningful words contrasting the six Cantonese tones on three base syllables/fɐn/, /jɐu/, and /wɐi/ (see Table 2). In addition, tones carried by the base syllable /ŋа/ served as the masking items in the discrimination task for both low and high variation conditions (for details see Procedure below). One female native Cantonese speaker was recorded reading aloud these words in a carrier sentence, 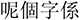/li55 ko33 tsi22 hɐi22/ (“This word is”) for six times. For each word, one clearly produced token was selected and segmented out of the carrier sentence. All selected words were normalized in duration to 620 ms using PSOLA, which is close to the mean of all selected tokens, and in mean acoustic intensity to 60 dB using Praat (Boersma and Weenink, 2014). The duration normalization was made as a whole unit, preserving the overall acoustic features.

**TABLE 2:** The five sets of syllables used in the experiment.

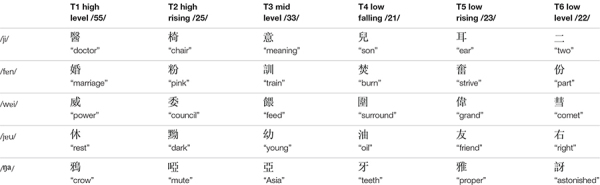

The non-speech tone stimuli were pure tone analogs of the stimuli that were used in the low variation condition. A 620-ms pure tone sound was first generated using Praat, and then a total of 12 F0 contours extracted from the syllable /ji/ and /ŋа/ were superimposed on the pure tone sound, generating 12 pure tone stimuli. The mean acoustic intensity of the pure tone sounds was 75 dB.

### Procedure

In each stimulus condition, there were an identification task and a discrimination task. The design of the identification task was adopted from the Bergen procedure (Hugdahl, 1995; Jia et al., 2013). In each trial, there was a dichotic pair presented to the two ears simultaneously. Some pairs were of the same tones, and some pairs were of different ones. Level and contour tones were presented in separate blocks. Both level and contour tones were examined in order to obtain a more comprehensive picture of lexical tone processing with more generalizable results. This factor was not included in the analysis for the reason that we do not have predictions about group differences in ear preference patterns in relation to this factor. For the level tones, there were three same pairs (T1-T1, T3-T3, and T6-T6) and six different tone pairs (T1-T3, T1-T6, T3-T6, T3-T1, T6-T1, and T6-T3). For the contour tones, there were also three same pairs (T2-T2, T4-T4, and T5-T5) and six different tone pairs (T2-T4, T2-T5, T4-T5, T4-T2, T5-T2, and T4-T5). In the low variation condition, the two items within each trial were always of the same syllable, e.g., /ji55/-/ji33/. In the non-speech tone condition, all the stimuli were pure tones that bore the same F0 trajectories as the stimuli used in the low variation condition. In the high variation condition, the two items in the dichotic pairs were always of different base syllables, for example, /fɐn55/-/jɐu33/. The subjects were asked to maintain equal attention to both ears and report the most clearly heard tone by pressing the button on the keyboard, i.e., 1–6, which indicate Tone 1 to Tone 6, respectively. The response had to be made within 5 s. There were six blocks in total in the identification task (2 tone types × 3 stimulus types). The three same tone pairs were repeated six times, and the six different pairs were also repeated six times, generating a total of 54 trials in each block. In the identification task, we did not balance the number of same and different pairs, for the reason that identical tones were presented to both ears in same pairs, which did not probe dichotic listening and was not our primary interest.

The design of the discrimination task followed those of Brancucci et al. (2008) and Jia et al. (2013). Different from the identification task, subjects were presented with two dichotic pairs with focused attention, that is, subjects were instructed to direct their attention to one testing ear. This paradigm guided the subjects to control the direction of attention (i.e., fluctuations of attention from one ear to the other are minimized) and was proven to be an effective paradigm to detect a consistent and reliable laterality effect (Brancucci and San Martini, 1999, 2003; Brancucci et al., 2008). In this paradigm, a dichotic pair composed of the target and the mask was first presented simultaneously, followed by a second dichotic pair composed of the probe and the mask. The target and probe were presented at the same ear that was the testing ear, and the two masking stimuli were always identical. The task was to judge whether the target-probe presented sequentially in the testing ear was the same or different. The subjects were required to respond within 3 s.

In the current study, in the non-speech tone condition, the masking items were pure tone stimuli which carried the same F0 trajectories with the syllable /ŋа/, and the target-probe items were pure tone stimuli which carried the same F0 trajectories with the syllable /ji/. In the low and high variation conditions, the masking items were words based on the syllable /ŋа/, and the carrying syllable(s) for the target-probe items was /ji/ for the low variation condition, and were /fɐn/, /jɐu/, and /wɐi/ for the high variation condition. Again, note that in the high variation condition, each target-probe tone pair was always associated with different syllables. If we apply all the possible syllable pairs within one block, the experiment would be too long and cause fatigue. In order to control the length of the experiment, two sets of syllables for the target-probe pairs in this condition were used. Set A consisted of three syllable pairs (/fɐn/-/jɐu/, /wɐi/-/fɐn/, /jɐu/-/wɐi/) and set B included the same syllable pairs in reversed order (/jɐu/-/fɐn/, /fɐn/-/wɐi/, /wɐi/-/jɐu/). Half of the subjects in each group were randomly assigned to set A, and the other half to set B. Like in the identification task, there were three same pairs (T1-T1, T3-T3, and T6-T6) and six different tone pairs (T1-T3, T1-T6, T3-T6, T3-T1, T6-T1, and T6-T3) for the level tones, and another set of three same pairs (T2-T2, T4-T4, and T5-T5) and six different tone pairs (T2-T4, T2-T5, T4-T5, T4-T2, T5-T2, and T4-T5) for the contour tones. The same pairs were repeated six times, and different pairs were repeated twice, creating equal numbers of same and different pairs. There were 12 blocks in total (2 ears × 2 tone types × 3 stimulus types).

All subjects completed the identification task first, followed by the discrimination task, for the reason that the identification task did not direct attention to one ear. Practice sessions were provided before both identification and discrimination tasks to familiarize the subjects with the procedure. The presentation order of blocks in each task was counterbalanced across subjects within the group as much as possible, and kept identical between amusics and matched controls. Stimulus presentation and data recording were implemented by E-prime 1.0.

### Data Analysis

For the identification task, accuracy and response time (RT) were analyzed. Identification accuracy was computed as the relative portion of the correct responses in each ear. Take one trial as an example, the right ear was presented with T1 and the left ear was presented with T3; if a participant’s response was T1, this trial was considered as correct in the right ear, and if the response was T3, it was considered as correct in the left ear. If the response was neither T1 nor T3, it was deemed as incorrect. To compare the accuracy of amusics and controls, linear mixed-effects models were fitted with *group* (amusics and controls), *stimulus type* (non-speech tone, low variation and high variation), and *ear* (left and right) as three fixed effects, and with *subject* as a random effect; two-way and three-way interactions were also included as fixed effects in the models. In order to test the significance of the fixed effects, a simple model (m_0) was first fitted, and the fixed effects and interactions, such as *group*, *stimulus type, and ear*, were added to the model consecutively. The model with a fixed effect (e.g., *group*) was compared with a baseline model without it. Models were compared by likelihood ratio tests and *p*-values were obtained from those tests.

Identification RT was measured from the offset of the stimuli to the time that a response was made. Trials with null responses were excluded from analysis. Incorrect trials were also discarded for the reasons that for the incorrect trial, it is impossible to know whether it was identified based on information from the left ear or the right ear. The RT data were first log-transformed to avoid the non-normality, presence of outliers, or unequal variances, and then linear mixed-effects models were fitted with *group* (amusics and controls), *stimulus type* (non-speech tone, low variation and high variation), and *ear* (left and right) as three fixed effects, and with *subject* as a random effect; two-way and three-way interactions were also included as fixed effects in the models. The procedure and tests for model comparisons are the same as those described above.

For the accuracy analysis in the discrimination task, generalized mixed-effects models were fitted on the responses to each trial (correct response was coded as “1” and incorrect response was coded as “0”). The fixed effects were *group* (amusics and controls), *stimulus type* (non-speech tone, low variation and high variation), and *ear* (left and right), and *subject* was input as a random effect; two-way and three-way interactions were also included as fixed effects in the models. The procedure and tests for model comparisons are the same as those described above.

RT in the discrimination task was measured from the offset of the second stimulus in a pair to the time that a response was made. The trials with null response were excluded from the analysis. Linear mixed-effects models were fitted on the log-transformed RT data. The procedures were the same as described above in the identification accuracy and RT analysis.

The analyses in this paper were all performed with R (R Core Team, 2014), using the *lme4* package (Bates et al., 2012), the *lmtest* package (Zeileis and Hothorn, 2002), and the *lsmeans* package (Lenth, 2016). Where *post hoc* comparisons were conducted within each fixed effect, for instance, to explore the main effect of stimulus type, “*glht*” function in the “*multcomp*” package (Hothorn et al., 2008) was used.

## Results

**FIGURE 1:**
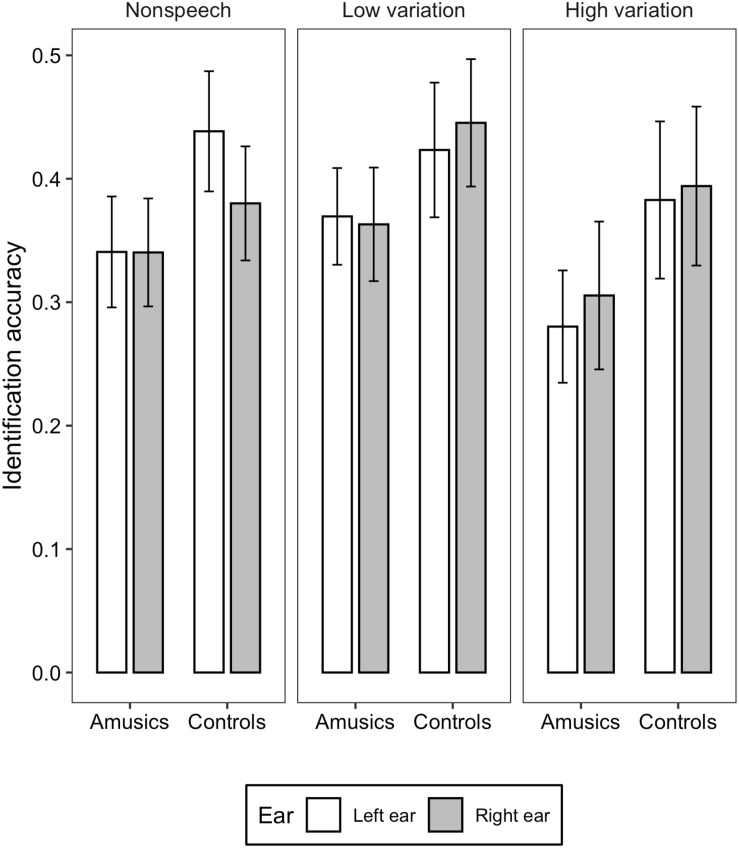
Bar charts showing the identification accuracy (means and CIs) in each ear for amusic and control participants in the three stimulus conditions: Non-speech, Low variation, and High variation.

**FIGURE 2:**
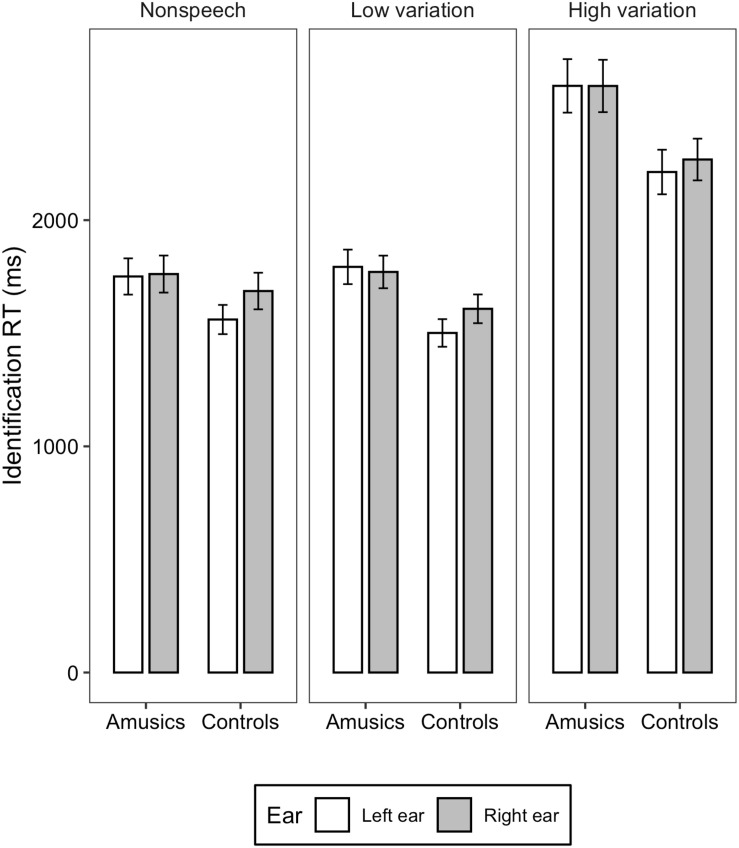
Bar charts showing the identification RT (means and CIs) for amusic and control participants in the three stimulus conditions: Non-speech, Low variation, and High variation.

**FIGURE 3:**
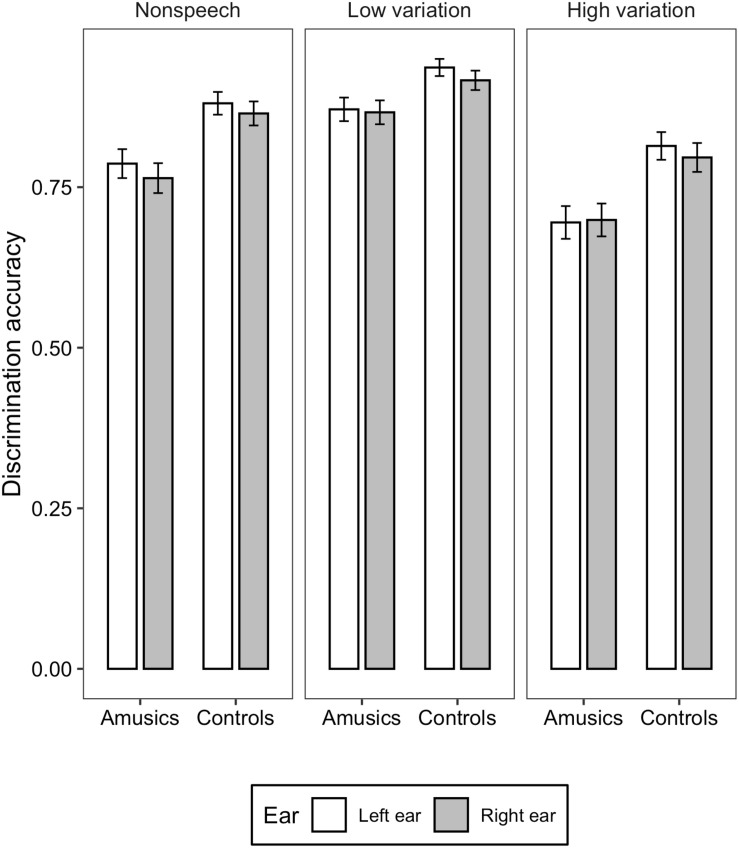
Bar charts showing the discrimination accuracy (means and CIs) in each ear for amusic and control participants in the three stimulus conditions: Non-speech, Low variation, and High variation.

**FIGURE 4:**
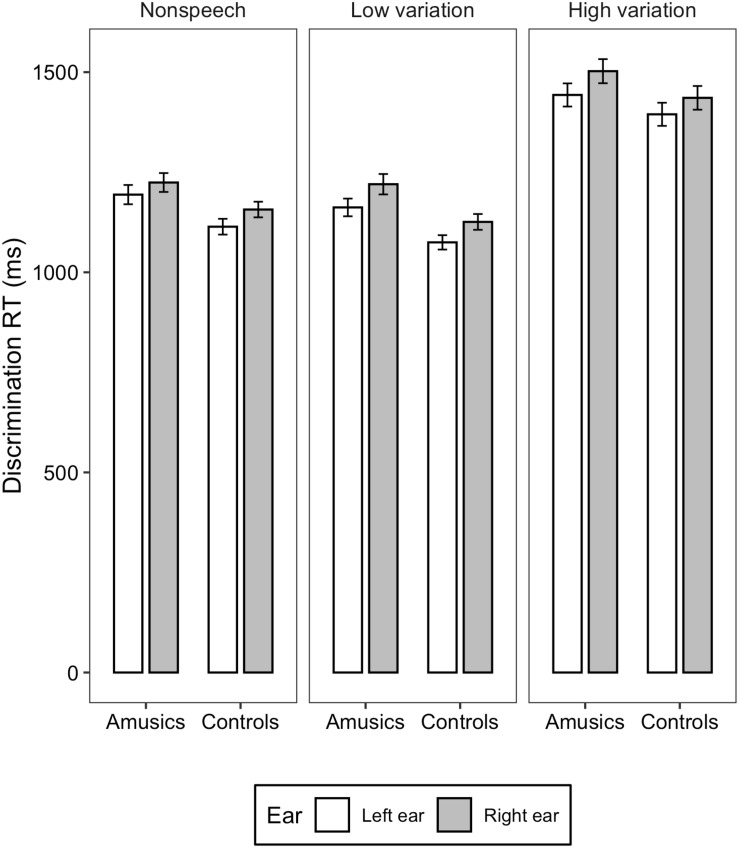
Bar charts showing the discrimination RT (means and CIs) in each ear for amusic and control participants in the three stimulus conditions: Non-speech, Low variation, and High variation.

Figures 1, 2 illustrate the accuracy and RT of the identification task; Figures 3, 4 display the accuracy and RT of the discrimination task.

For the identification accuracy, liner mixed-effects models found significant main effects of *group* [χ^2^(1) = 15.905, *p* < 0.001] and *stimulus type* [χ^2^(2) = 11.723, *p* < 0.01]. Amusics performed significantly worse than controls (*M* = 0.333, *SD* = 0.140 vs. *M* = 0.410, *SD* = 0.164). *Post hoc* analysis revealed that the accuracy in the high variation condition was significantly lower than that in the low variation condition (*z* = −3.428, *p* < 0.001; *M* = 0.340, *SD* = 0.178 vs. *M* = 0.400, *SD* = 0.145); the differences between the low variation and non-speech tone condition (*z* = - 1.461, *p* = 0.309; *M* = 0.400, *SD* = 0. 0.145 vs. *M* = 0.374, *SD* = 0.140), and between the high variation and non-speech tone condition were not significant (*z* = −1.967, *p* = 0.120; *M* = 0.340, *SD* = 0.178 vs. 0.374, *SD* = 0.140). The effect of *ear* [χ^2^(2) = 0.006, *p* = 0.938], the two-way interaction between *group* and *ear* [χ^2^(2) = 0.253, *p* = 0.613], between *ear* and *stimulus type* [χ^2^(2) = 2.087, *p* = 0.352], between *group* and *stimulus type* [χ^2^(2) = 0.821, *p* = 0.663], and three-way interaction among *group*, *ear*, and s*timulus type* [χ^2^(2) = 1.564, *p* = 0.457] were all not significant.

For the identification RT, linear mixed-effects models fitted on the transformed RT found significant main effects of *group* [χ^2^(1) = 5.0541, *p* = 0.02), *ear* (χ^2^(1) = 4.1897, *p* = 0.04], and *stimulus type* [χ^2^(2) = 637.9, *p* < 0.001]. The identification RT was significantly shorter in the control group than in the amusia group (*M* = 1840, *SD* = 617 vs. *M* = 2078, *SD* = 647). The left ear showed significantly shorter RT than the right ear (*M* = 1822, *SD* = 623 vs. *M* = 1839, *SD* = 664). Concerning the effect of *stimulus type*, RT elicited in the high variation condition was significantly longer than that in the low variation and non-speech conditions (*p*s < 0.001; *M* = 2470, *SD* = 665 vs. *M* = 1680, *SD* = 412 vs. *M* = 1725, *SD* = 485), whereas the difference between the low variation and non-speech conditions was not significant (*z* = 0.038, *p* = 0.999; *M* = 1680, *SD* = 412 vs. *M* = 1725, *SD* = 485). The two-way interaction between *group* and *ear* [χ^2^(2) = 2.012, *p* = 0.156], between *ear* and *stimulus type* [χ^2^(2) = 0.048, *p* = 0.976], between *group* and *stimulus type* [χ^2^(2) = 3.832, *p* = 0.147], and three-way interaction among *group*, *ear*, and s*timulus type* [χ^2^(2) = 0.789, *p* = 0.674] were all not significant.

For the discrimination accuracy, generalized mixed-effects models revealed significant main effects of *group* [χ^2^(1) = 14.248, *p* < 0.001], *ear* [χ^2^(1) = 4.297, *p* = 0.03], and *stimulus type* [χ^2^(2) = 402.71, *p* < 0.001]. Amusics demonstrated significantly lower discrimination accuracy than controls (*M* = 0.78, *SD* = 0.13 vs. *M* = 0.87, *SD* = 0.11). Accuracy in the left ear was significantly higher than the right ear (*M* = 0.83, *SD* = 0.12 vs. *M* = 0.81, *SD* = 0.13). Discrimination accuracy differences among the three stimulus types were all significant, with the low variation condition eliciting the highest accuracy score, followed by the non-speech condition, and finally the high variation condition (*p*s < 0.001; *M* = 0.89, *SD* = 0.09 vs. *M* = 0.82, *SD* = 0.09 vs. *M* = 0.75, *SD* = 0.11). The two-way interaction between *group* and *ear* [χ^2^(2) = 1.589, *p* = 0.207], between *ear* and *stimulus type* [χ^2^(2) = 1.254, *p* = 0.534], between *group* and *stimulus type* [χ^2^(2) = 0.908, *p* = 0.635], and three-way interaction among *group*, *ear*, and s*timulus type* [χ^2^(2) = 0.998, *p* = 0.607] were all not significant.

For the discrimination RT, linear mixed-effects models found significant main effects of *ear* [χ^2^(1) = 37.95, *p* = 0.03] and *stimulus type* [χ^2^(2) = 1408, *p* < 0.001]. RT elicited in the left ear was significantly shorter than in the right ear (*M* = 1233, *SD* = 281 vs. *M* = 1282, *SD* = 292). RT differences among the three stimulus types were all significant (*p*s < 0.001), with the high variation condition eliciting the longest RT, followed by the non-speech and low variation condition (*M* = 1451, *SD* = 248 vs. *M* = 1173, *SD* = 218 vs. *M* = 1146, *SD* = 217). The two-way interaction between *group* and *ear* [χ^2^(2) = 0.330, *p* = 0.565], between *ear* and *stimulus type* [χ^2^(2) = 0.006, *p* = 0.996], between *group* and *stimulus type* [χ^2^(2) = 2.700, *p* = 0.259], and three-way interaction among *group*, *ear*, and s*timulus type* [χ^2^(2) = 1.212, *p* = 0.545] were all not significant.

For all the four sets of analyses reported above, the mixed-effects models did not obtain significant interactions between *group* and *ear*. To further test this null effect (H0: no significant two-way interaction between *group* and *ear*), we conducted Bayesian two-way ANOVA (*group* by *ear*) with JASP (JASP Team, 2020). The dependent variables were the identification accuracy, the identification RT, the discrimination accuracy, and the discrimination RT, respectively. For the identification accuracy, Bayesian analysis showed that BF_01_ = 59.29, which means that the data are approximately 59 times more likely to occur under the H0 (null hypothesis: no interaction between *group* and *ear*) than under the H1 (the alternative hypothesis). The error percentage is 3.809%, which reflects the stability of the numerical algorithm that was used to obtain the results. This result indicates strong evidence in favor of the null hypothesis. Similar patterns were obtained for the identification RT (BF_01_ = 28.126, error percentage = 1.346%), discrimination accuracy (BF_01_ = 21.590, error percentage = 1.297), and discrimination RT (BF_01_ = 13.996, error percentage = 1.411). Note that all the Bayes Factors reported here concern the non-significant *group* by *ear* interaction. Altogether, these Bayes factors provided additional support for the null interaction between group and ear reported above.

To summarize, overall, amusics performed worse than the controls in both tone identification and discrimination tasks in the dichotic listening settings, indicating inferior abilities in lexical tone perception in the amusic individuals. The interaction between *group* and *ear* was not significant, and additional Bayesian analyses provided strong evidence supporting the null interaction between group and ear. For both groups, in the discrimination task, an LEA was found in terms of both accuracy and RT. In the identification task, where more phonological processing was required, the shorter RT in the left ear might somehow still suggest a pattern of LEA, but the identification accuracy didn’t show a significant ear preference, suggesting a shift from LEA to more bilateral processing of lexical tones in the identification task. Concerning the effects of *stimulus type*, stimuli with high syllable variations were more difficult to identify or discriminate than the other two types of stimuli, however, no interaction was found between *stimulus type* and *ear*.

## Discussion

A number of studies have consistently reported that individuals with amusia showed deficits in lexical tone perception in both phonetic and phonological levels (Nan et al., 2010; Tillmann et al., 2011b; Jiang et al., 2012b; Liu et al., 2012; Zhang et al., 2017b; Shao et al., 2019). However, it is still unclear how these deficits in different processing levels influence the ear preference/brain lateralization of pitch and lexical tone perception in the amusic population. In the current study, we investigated the brain specialization of pitch and lexical tone perception in amusics and typical listeners by adopting a dichotic listening paradigm in three different stimulus conditions. Amusics demonstrated a similar tendency of brain lateralization as the typical listeners, but showed overall degraded performance in the dichotic listening of non-speech tones and lexical tones.

Previously, several hypotheses regarding the brain specialization patterns in lexical tone perception have been proposed (Wang et al., 2001; Luo et al., 2006; Jia et al., 2013). On the one hand, as lexical tones are pitch modulations, when attention was directed to the auditory/phonetic level of pitch processing, as in pure tone or hum conditions or when non-native listeners perceive lexical tones, it primarily involves the RH, showing an LEA. On the other hand, if lexical tones are perceived as distinctive features in a more linguistically relevant task that involves phonological computing in native listeners, they are predominantly processed by the LH, showing bilateral processing or an REA. In the present study, we found that for both groups, an evident LEA was found in the discrimination task as reflected by the discrimination accuracy and RT. In contrast, in the identification task, although the identification RT was shorter in the left ear (1,822 ms for the left ear and 1,839 ms for the right ear), which might somehow suggest an LEA, no lateralized difference was obtained in terms of the identification accuracy, suggesting a bilateral pattern of lexical tone processing. Taken together, the results of the identification task indicate that the LEA in the discrimination task may become unstable in the phonologically demanding identification task and more LH was involved. In other words, the recognition of lexical tone categories could induce a shift from the LEA to more bilateral processing. This point is further elaborated on below.

We propose that the different patterns revealed by identification and discrimination tasks reflect processing differences. The identification task involves tone categorization and measures higher-level phonological processing of lexical tones. In the discrimination task, it might primarily tap into phonetic processing of lexical tones or auditory comparison of non-speech tones, as the task mainly requires the comparison of the acoustic features of two tokens; for naïve speakers without tonal knowledge, they can still perform the discrimination task. Therefore, discrimination and identification have been considered to measure different aspects of perceptual abilities (Iverson et al., 2012), eliciting different brain lateralization patterns. As a result, a shift from the LEA to bilateral processing was observed in the identification task that involves more phonological processing, in contrast to a strong and consistent LEA in the discrimination task in the current study. The bilateral pattern is also consistent with the finding of a recent meta-analysis on the functional brain activity of lexical tone perception, which revealed that both hemispheres are involved in the processing of lexical tones in native tonal language speakers (Liang and Du, 2018).

Our findings were partially consistent with the results of Jia et al. (2013), who reported an overall LEA in the discrimination task irrespective of stimulus types in Cantonese speakers. Nonetheless, we obtained a bilateral pattern in the identification task in terms of the accuracy, which somewhat diverged from the LEA (RH advantage) that (Jia et al., 2013) reported in terms of both accuracy and RT in the identification task. Despite the discrepancy, note that the patterns in the current study are consistent with our prediction of greater involvement of the LH in the phonologically demanding identification task, as explained above. It is possible that our design included a more demanding condition, i.e., the high syllable variation condition, which may have encouraged more phonological processing. As a matter of fact, Jia et al. (2013) also predicted that discrimination and identification may elicit different ear preference patterns, as these two tasks involve linguistic processing to different extents.

Compared with previous dichotic listening studies on other tonal languages, our results are in agreement with Baudoin-Chial (1986), who observed bilateral effects of lexical tone perception in Mandarin speakers, but differed from those that found an REA in lexical tone perception (Van Lancker and Fromkin, 1973, 1978; Moen, 1993; Wang et al., 2001). Two possible factors may contribute to the different results, namely native language experience and alphabetic literacy. As Jia et al. (2013) argued, for native Cantonese speakers, the unique tonal system may contribute to the RH advantage in perceiving lexical tones. The tonal space of Cantonese is denser than that of Mandarin (Bauer and Benedict, 2011; Peng, 2006; Peng et al., 2012; Matthews and Yip, 2013). Perception of the six tones in Cantonese relies on both pitch height and pitch direction, and Cantonese speakers may be urged to make use of finer acoustic details to distinguish Cantonese tones. For instance, discriminating the three level tones in Cantonese relies on the comparison of the relative pitch height differences, and the analysis of subtle acoustic differences is known to be predominantly processed by the RH. A second factor is the lack of systematic alphabetic literacy of spoken Cantonese in Cantonese speakers in Hong Kong. Previous studies have shown that alphabetic literacy can enhance phonological awareness, including tone awareness, and restructure the phonological network in the LH (Morais et al., 1979; Cheung et al., 2001; Shu et al., 2008; Dehaene et al., 2010; Brennan et al., 2013). In light of this finding, it is likely that more LH activation is involved in lexical tone perception among native Mandarin speakers, which leads to the observed REA, since the majority of Mandarin speakers in the Mainland have received systematic instruction of *Pinyin*, an alphabetic script of spoken Chinese. In contrast, many Cantonese speakers in Hong Kong are only literate in logographic Chinese, and have no systematic knowledge of an alphabetic script of Cantonese. Indeed, it is often difficult to ask Cantonese speakers to recognize/label the tone (e.g., Tone 1, Tone 2, etc.) without some training or practice, whereas asking them to identify the spoken words that carry tonal contrasts is more intuitive.

The current study did not obtain a significant interaction between *stimulus type* and *ear*, which is consistent with Jia et al. (2013) but not in line with Mei et al. (2020). One plausible explanation could be that our participants lack alphabetic literacy of spoken Cantonese, as discussed above. When investigating a group of native Cantonese speakers who have received training in *Jyutping*, an alphabetic code of Cantonese, the results revealed that the LEA in the discrimination task only stand in the non-speech and low variation condition, but not in the high variation condition (i.e., a *stimulus type* × *ear* interaction) (Shao et al., under review). These findings provided some evidence that the interaction between *stimulus type* and *ear* could be partially influenced by the speakers’ native language skills (i.e., alphabetic literacy).

Regardless of ear preference, amusics showed overall lower scores in both identification and discrimination tasks than controls in all stimulus types, suggesting that amusic individuals are impaired in lexical tone perception in dichotic listening settings. These results confirmed that amusics are generally impaired in tone perception (Nan et al., 2010; Liu et al., 2016; Shao et al., 2016, 2019; Zhang et al., 2017b). For example, Shao et al. (2019) found that Cantonese-speaking amusics demonstrated inferior performance under both conditions when tones were associated with the same syllable and when tones were associated with different syllables. Together with these findings, our results further proved that when the tones were presented in a dichotic manner (identification task), or when the attention was intentionally directed to a target ear (discrimination task), amusics showed consistently degraded performance, under all the task conditions (non-speech, low syllable variation, and high syllable variation).

Intriguingly, although amusics showed degraded performance, there is no clear evidence that their ear preference patterns were different from typical controls. This finding may suggest that the impairment in pitch/lexical tone processing is likely to have no direct impact on ear preference. Previous neuroimaging studies have reported neural impairments in a RH frontotemporal network including the IFG and STG in amusia (Hyde et al., 2011; Albouy et al., 2013; Chen and Yuan, 2016; Wang et al., 2017; Zhang et al., 2017a). Additionally, a number of EEG studies suggested that the amusics’ brain showed abnormal responses during attentive processing of musical pitch and lexical tones with small pitch differences (Peretz et al., 2009; Moreau et al., 2013; Zendel et al., 2015; Zhang and Shao, 2018). In contrast to these studies, our results suggested that despite impaired neural activities during musical pitch and lexical tone processing, there is no direct evidence that ear preference in lexical tone perception was much affected in amusic individuals. One possible explanation is that unlike second language learners, amusics have established routines in processing native lexical tones, which may facilitate the maintaining of similar ear preference patterns despite their deficits in phonetic and phonological processing of lexical tones. It should also be acknowledged that as a behavioral paradigm, dichotic listening could reveal the final outcome of ear preference, but it lacks the spatial and temporal resolution to reveal the precise brain lateralization pattern and its dynamic change over time like EEG and fMRI. One last possibility is that there might exist complex neural compensational mechanisms in the amusical brain, which are not well understood currently, leading to similar ear preference patterns in amusics in the final behavioral outcome. Future neuroimaging studies should investigate these issues.

Lastly, both controls and amusics tended to benefit from the speech stimuli more than the non-speech stimuli, showing better performance in the low syllable variation condition than the non-speech condition in the discrimination task (as indexed by the discrimination accuracy and RT), and no significant difference between the two types of stimuli in the identification task. These findings may shed some light on the effect of stimulus type on speech processing in amusia. Previously, some findings suggested that non-tonal language speakers without musical training showed reduced sensitivity to pitch differences in lexical tones compared with non-linguistic pitch, while musicians with absolute pitch demonstrated equally good performance across all types of pitch stimuli (Burnham et al., 2015). Concerning the amusic population, some studies suggested that amusics can perform better in natural speech stimuli than non-speech stimuli (Ayotte et al., 2002; Jiang et al., 2010; Tillmann et al., 2011b). For example, Tillmann et al. (2011b) found that pitch discrimination was better in the spoken syllables than the acoustically matched non-speech tones for amusics, whereas other studies demonstrated opposite results (Liu et al., 2010), and a third line of research showed mixed patterns (Liu et al., 2012). The current findings confirmed that linguistic information might facilitate pitch perception in speech stimuli relative to non-linguistic tone analogs in both amusics and controls. These patterns are different from those from Burnham et al. (2015), probably because of the listeners’ native language background. Tonal language speakers in the current study may be better able to make use of pitch cues in the speech stimuli, as they are meaningful words. Moreover, this facilitation effect of speech stimuli appears to depend on the type of task. It is possible that discriminating two stimuli within each trial relies on short-term pitch memory, which is known to be impaired in amusics (Williamson and Stewart, 2010; Albouy et al., 2013), and as a result meaningful speech stimuli may alleviate the burden of short-term pitch memory to some extent. Another possible reason for the lack of stimulus effects in the identification task is that the subjects were asked to label the non-speech with lexical tone categories, and for this reason might process the non-speech stimuli in a similar manner as lexical tones.

## Conclusion

To conclude, our results revealed an LEA in the discrimination of lexical tones as indexed by the discrimination accuracy and RT and reduced LEA or bilateral processing in tone identification as indexed by the identification RT and accuracy. The performance of amusic individuals was overall degraded, suggesting impaired lexical tone processing in dichotic settings. However, the ear difference between amusics and controls was not significant, implying that so far there was no evidence to indicate that amusics showed different ear preference patterns from controls. The Bayes factors provided additional strong support for these null effects. These results temporarily indicated that although amusics showed abnormal brain responses to pitch/lexical tone, these impairments might not directly influence their ear preference. Future studies should further verify this finding with a larger sample size. These findings broadened our understanding of the deficits in amusia and also shed some light on the brain specialization for pitch and lexical tone perception in amusic individuals.

## Data Availability Statement

The datasets generated for this study are available on request to the corresponding author.

## Ethics Statement

The studies involving human participants were reviewed and approved by the Human Subjects Ethics Sub-Committee of The Hong Kong Polytechnic University. The patients/participants provided their written informed consent to participate in this study.

## Author Contributions

JS: conceptualization, methodology, formal analysis, and writing - original draft. CZ: conceptualization, writing - review and editing, and funding acquisition. Both authors contributed to the article and approved the submitted version.

## Conflict of Interest

The authors declare that the research was conducted in the absence of any commercial or financial relationships that could be construed as a potential conflict of interest.
